# Ocular ultrasonography focused on the posterior eye segment: what radiologists should know

**DOI:** 10.1007/s13244-016-0471-z

**Published:** 2016-02-24

**Authors:** Marcela De La Hoz Polo, Anna Torramilans Lluís, Oscar Pozuelo Segura, Albert Anguera Bosque, Catalina Esmerado Appiani, Josep Maria Caminal Mitjana

**Affiliations:** Radiology Department, Hospital Sant Pau i Santa Tecla, Rambla Vella 14, 43003 Tarragona, Spain; Hospital El Vendrell, Carretera de Barcelona, s/n, 43700 El Vendrell, Spain; Radiology Department, Hospital de Viladecans, Viladecans, Spain; Ophthalmology Department, Hospital de Viladecans, Viladecans, Spain; Ophthalmology Department, Hospital Universitari de Bellvitge, L’Hospitalet de Llobregat, Spain

**Keywords:** Ultrasound, Posterior eye segment, Retina, Vitreous body, Choroid

## Abstract

**Abstract:**

Ocular B-mode ultrasonography (US) is an important adjuvant for the clinical assessment of a variety of ocular diseases. When ophthalmoscopy is not possible, mainly due to opacification of the transparent media (e.g., mature cataract or vitreous haemorrhage), US can guide the ophthalmologist in diagnosing disease and choosing treatment. The superficial location and cystic structure of the eye make US ideal for imaging of the eye. Moreover, dynamic study helps distinguish between various conditions that would otherwise be difficult to differentiate in some clinical setting, such as vitreous, retinal, and choroidal detachment. US is also good technique for detecting other pathologic conditions such as lens dislocation, vitreous haemorrhage, asteroid hyalosis, optic disc drusen, and tumors (e.g., choroidal melanoma, metastases, hemangioma). An understanding of the basic anatomy of the eye, the US technique, and common entities that affect the ocular globe will allow radiologists to offer this valuable imaging modality to patients and referring clinicians. This article focuses on the US anatomy and pathologic conditions that affect the posterior ocular segment.

***Teaching points*:**

• US is specially indicated when ocular fundus cannot be assessed on ophthalmoscopy.

• Multipurpose equipment with high-frequency transducers is optimal for imaging the eye.

• Ultrasound can reliably depict ocular anatomy and pathology as detachments and tumours.

• Dynamic examination is vital for distinguishing certain pathologic conditions as detachments.

**Electronic supplementary material:**

The online version of this article (doi:10.1007/s13244-016-0471-z) contains supplementary material, which is available to authorized users.

## Introduction

Ocular US has long been the province of ophthalmologists, often using dedicated equipment [1]. However, radiologists are becoming increasingly involved, using general (multipurpose) ultrasound equipment with high-frequency small parts probes.

The cornea, anterior chamber, iris, posterior chamber and lens rarely require US, because they can be properly evaluated by clinical inspection, ophthalmoscopy, slit-lamp examination, and US biomicroscopy using frequencies up to 50 MHz [2, 3]. Nevertheless, any condition that causes opacification of the light-conducting media may obscure visualization of the posterior segment of the globe at clinical examination, thus requiring B-mode US to rule out retinal, vitreous, and choroidal detachments, tumours, and other pathologic conditions that affect the posterior segment of the eye. US can also provide useful additional information about disease detected in the ophthalmoscopic examination. It is the quickest and simplest method of imaging the eye; it is widely available, provides high-resolution images, and enables dynamic study. With appropriate training, qualified professionals can perform ocular US using a systematic study protocol.

Although computed tomography (CT) and magnetic resonance imaging (MRI) are very useful in many ocular and orbital conditions, they cannot scan in real time, have poorer spatial resolution, and have a limited role in the evaluation of the vitreous, retina and choroid.

This article will review the normal ocular anatomy on US, outline a systematic approach and study protocol for ocular US, and describe and illustrate US findings for pathologic conditions that affect the eyeball, especially the posterior segment (Table 1). The appropriate use of ocular US and correlation with other ophthalmologic diagnostic techniques enables a multidisciplinary approach to diagnosis, treatment planning, and follow-up.

**Table 1:** Summary of Ocular Pathologic Conditions Based on the Affected Structure

Ocular globe size and shape	
	Posterior staphyloma
	Phthisis bulbi
Lens	
	Cataracts
	Dislocation
Vitreous	
	Persistent hyperplastic primary vitreous
	Asteroid hyalosis
	Vitreous haemorrhage
	Posterior vitreous detachment
Retina	
	Retinal detachment
	Proliferative diabetic retinopathy
Choroid	
	Choroid detachment
	Choroidal tumors
Optic disc	
	Drusen
Post-surgical conditions	
	Pseudophakia
	Scleral buckle
	Silicone oil
	Perfluorocarbon liquids (PFCL)
	Intraocular air and gas

## Anatomy of the ocular globe

**Fig. 1:**
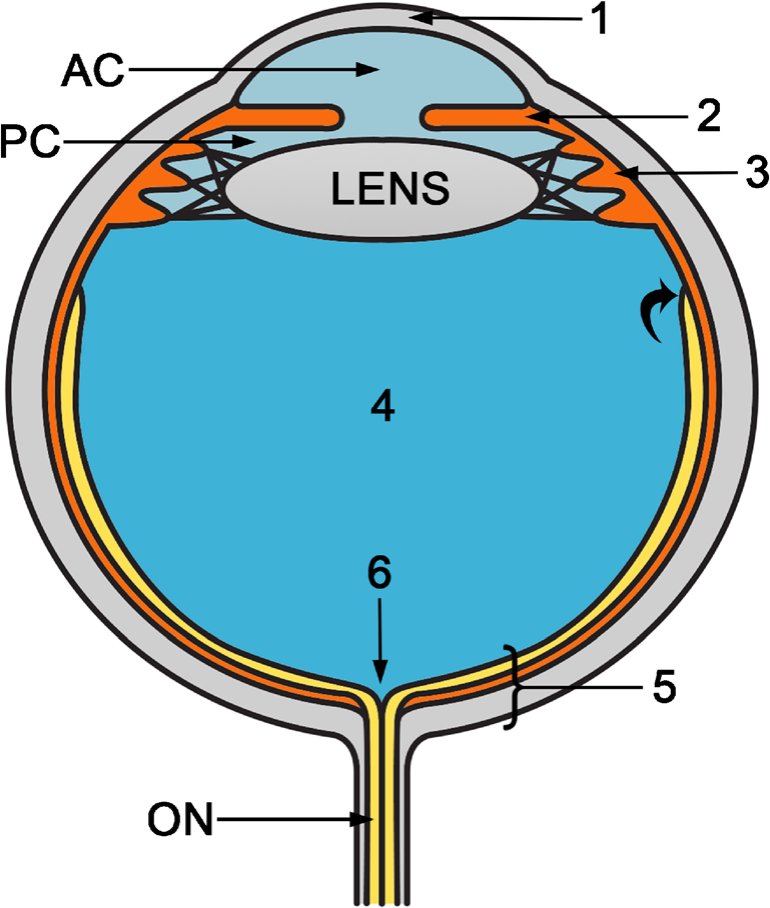
Diagram illustrating ocular anatomy. The anterior segment comprises the cornea (1), anterior chamber (AC), iris (2), ciliary body (3), lens, and posterior chamber (PC). The AC and PC are filled with aqueous humour. The lens is laterally attached to the ciliary body. The posterior segment comprises the vitreous chamber (4) and the posterior ocular wall (5), which is formed by the retina, choroid, and sclera (posterior RCS complex). The vitreous chamber is filled with vitreous humour, and its periphery is called the vitreous capsule or hyaloid. The retina anchors anteriorly at the ora serrata (*curved arrow*) and posteriorly at the optic disc (6). The choroid anchors anteriorly at the scleral spurs near the ciliary bodies and posteriorly near the exit foramina of the vortex veins (at some distance anterior to the optic disc). Behind the globe, the optic nerve (ON) is seen.

The globe lies in the anterior region of the orbit. It is surrounded by fat, but separated from it by a membranous sac, the capsule of Tenon. Its attachments include the corneoscleral junction anteriorly and the optic nerve posteriorly. Tenon’s capsule is pierced by the tendons of the extraocular muscle [4, 5]. The sclera and the cornea form the fibrous outermost layer; the vascular uveal tract, including the ciliary body anteriorly and the choroid posteriorly, forms the middle layer; and the retina forms the innermost, sensory layer. The lens is connected to the sclera by radially oriented zonular fibers. The lens divides the globe into an anterior and a posterior segment. The anterior segment contains the aqueous humour; it is formed by the cornea, anterior chamber, iris, posterior chamber, lens, and ciliary body. The posterior segment is filled with a gel-like substance called the vitreous humour, contained within the hyaloid membrane. The hyaloid membrane has two parts: the anterior part adheres to the posterior lens capsule, and the posterior part adheres to the internal limiting membrane of the retina. Normally, the vitreous is strongly adhered to the vitreous base, macula, margins of the optic disc, and retinal vessels. The posterior segment is also formed by the retina, choroid, and sclera (Fig. 1). In the normal eye, these three layers are adherent, but under certain pathologic conditions, they may separate and form potential spaces. The retina extends from the optic nerve to the ora serrata, the anterior-most part of the retina, which extends approximately three-quarters of the way from the optic nerve to the iris plane. The choroid is fixed at the scleral spurs just anterior to the ora serrata and posteriorly at some distance anterior to the optic disc at the exit foramina of the vortex veins. Knowledge about the attachment points of the various layers of the inner ocular wall is critical to understanding ocular detachments [5]. Vessels lie within the orbital fat of the muscle cone. Centrally, the optic nerve sheath passes from the posterior globe to the brain. The optic nerve sheath is an extension of the dura mater and contains the optic nerve, central retinal artery, and vein [1, 4, 6, 7].

**Fig. 2:**
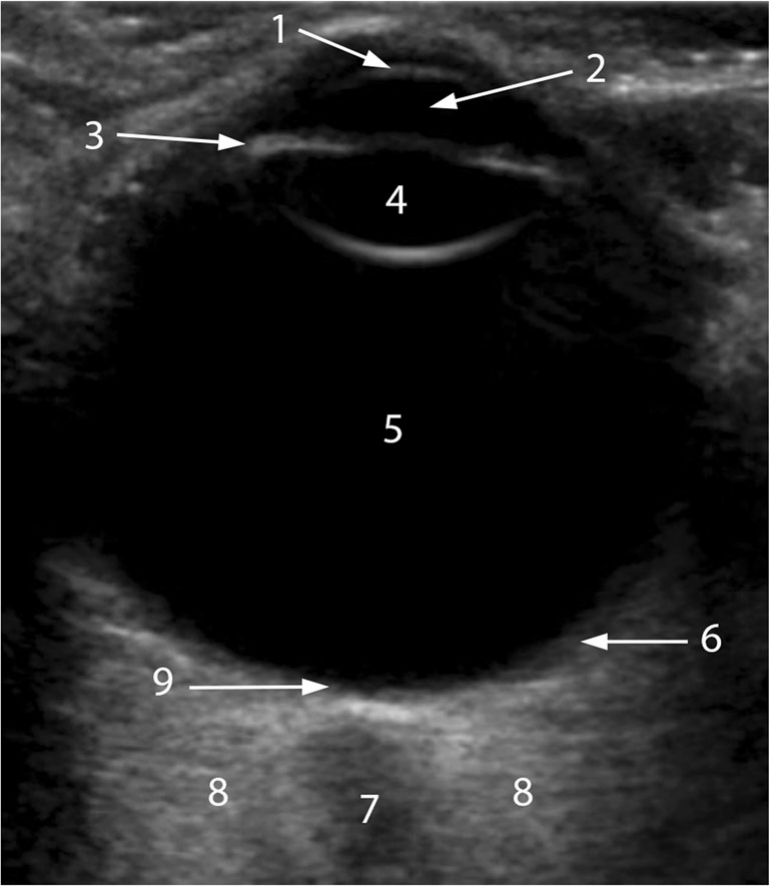
Sonographic appearance of the structures of the normal eye. The cornea (1) is visualized as the most superficial echogenic curved line; the anterior chamber (2) is anechoic. The iris (3) appears as a thin echogenic line. The lens (4) is defined by anterior and posterior boundary echoes, but the lens itself is echo-free. The vitreous chamber (5) is filled with a clear gel-like substance that is normally echo-free, although the formation of spots and linear echoes with aging is considered normal. The RCS complex (6) forms the wall of the posterior ocular segment; it is seen as an echogenic concave line extending from the iris plane to the optic nerve (ON; 7). The ON is seen as a hypoechoic band surrounded by echogenic retrobulbar fat (8). The circular area where the ON connects to the retina is the optic disc or papilla (9)

On US examination, the cornea is the most superficial structure; it appears as a thin line that at times can be difficult to identify. The anterior chamber is the anechoic area that lies between the cornea and the iris. The lens is seen as an anechoic structure with thin anterior and posterior echogenic capsules, and the ciliary body is seen as a hypoechoic line on either side of the lens. The vitreous is an anechoic area posterior to the lens. The posterior wall, comprising the retina, choroid, and sclera (RCS complex), appears as a concave echogenic line that is interrupted by the optic disc or papilla. The optic nerve sheath is seen as a hypoechoic tubular structure extending away from the globe posteriorly (Fig. 2). The central retinal artery and vein, which supplies the inner two-thirds of the retina, and short posterior ciliary arteries are seen on colour Doppler US. The ophthalmic artery and superior ophthalmic vein can also be seen at the retrobulbar orbital fat [6–9].

**Fig. 3:**
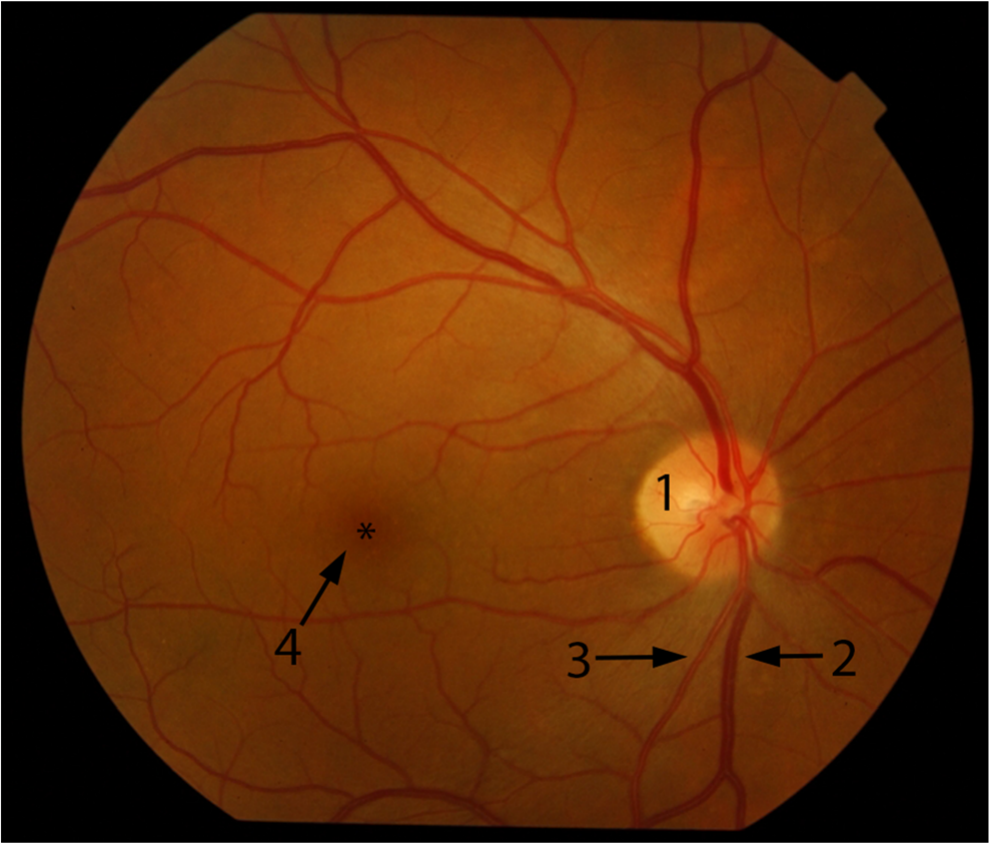
Normal fundoscopy of the right eye shows the oval optic disc (1); retinal veins (2) and arteries (3) radiate from the centre. The macula (4) is the round dark area lateral to the disc on the temporal side of the eye. The macula has a depressed spot, the fovea centralis (*asterisk*), which is the area of most acute vision

A normal ophthalmoscopic examination shows the oval optic disc with retinal vessels radiating from the centre; the retinal veins, which are wider than the arteries; the central retinal artery, which enters the optic nerve behind the eyeball and divides into superior and inferior branches; and the macula, which is seen as a round dark area lateral to the disc on the temporal side of the eye. The macula has a depressed spot, the fovea centralis, which is the area of most acute vision (Fig. 3) [10].

## Technique and study protocol

### Technique

**Fig. 4:**
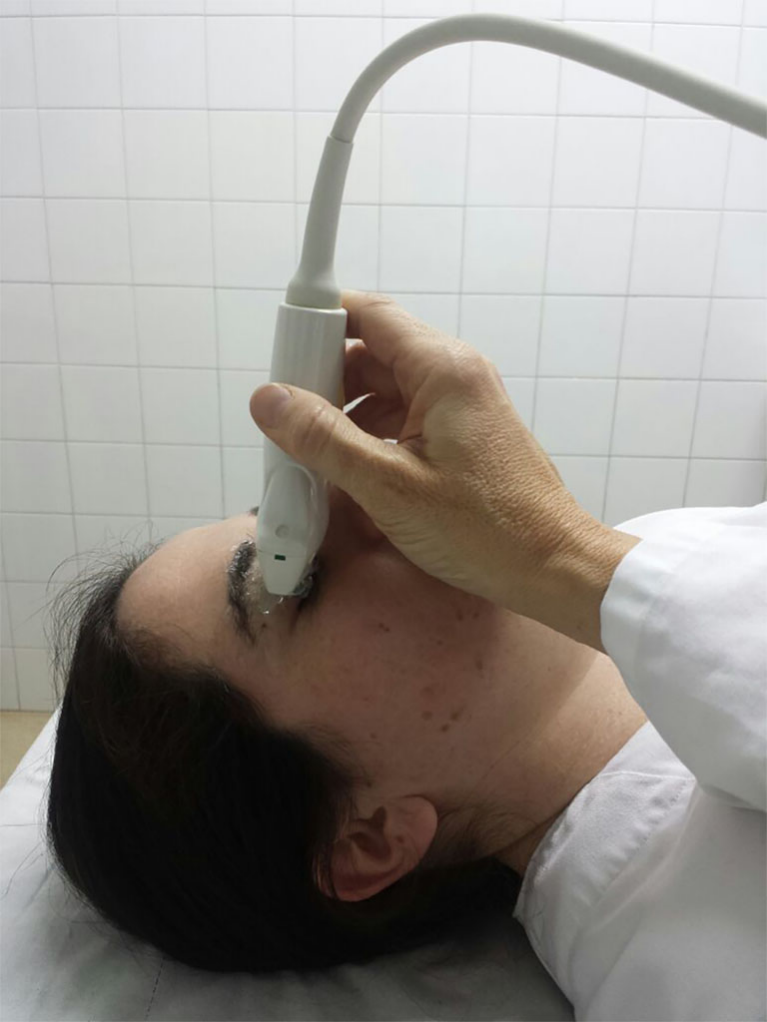
US technique. Photograph from a standard examination shows the position of the transducer to acquire axial images of the eye. Gel should be applied abundantly to allow better contact between the transducer and the eye

With the patient in the supine position, the eye is examined through the closed eyelid with high-frequency linear transducers, most commonly 7.5–13.0 MHz. Gel should be abundantly applied to the closed eyelid to allow better contact. The examination should be performed first in B-mode, and the focus, gain, and settings should be adjusted during the examination. The focus should be adjusted to the depth of the segment to be examined [11]. Greatly reducing the gain will show the walls of the globe and optic nerve sheath perfectly. Increasing the gain enables the contents of the vitreous body to be studied. Finally, adding colour and pulsed Doppler may be useful in some conditions [7, 12]. Low-flow settings and a small gate should be chosen for Doppler. The dynamic exam may be recorded, depending on the equipment, with a series of images or with video sequences (Fig. 4).

### Study protocol

The globe should be scanned in the neutral position and during gentle eye movements [6]. Before beginning the procedure, it is important to instruct patients regarding the ocular movements that will be required of them during the examination. The study should begin with axial sections from the upper to lower poles of the entire globe, and sagittal sections from the temporal to the nasal side. Oblique images are often useful. This multiplanar approach allows us to obtain a good 3-D mental image of the US findings [5, 8, 11]. It is helpful to obtain central images, aligning with the cornea, iris, lens, and optic disc, which ensures that the images from both eyes will be reproducible and useful for comparison and follow-up. The dynamic examination, in which patients are asked to move their eyes from right to left and up to down without opening their eyelids, is essential, especially for detecting diseases of the vitreous and detachment of the posterior vitreous, retina, and choroid [12]. Examination of the contralateral eye is recommended. It is important always to avoid exerting pressure on the eye, so as to prevent the anterior chamber from collapsing. This is especially important in ocular trauma to avoid rupturing of a previously injured eye wall and in postoperative patients [7]. While ocular trauma was once a contraindication for ocular US, nowadays there is evidence demonstrating the ability of US to safely diagnose closed- and open-globe injuries. Nevertheless, in patients with ocular trauma, clinical judgment would determine whether US should be performed, especially if a ruptured globe is suspected [4, 13, 14].

Colour Doppler is helpful in cases of suspected vascular abnormalities such as central retinal vessel occlusion, as well as in inflammatory diseases and tumours [9, 15].

## Pathologic US findings

### Alterations in size and shape of the ocular globe

The anteroposterior diameter of the ocular globe is approximately 22 to 25 mm in adolescents and adults [7, 16]. Obtaining accurate measurements in B mode requires specially calibrated US scanners and a meticulous approach.

**Fig. 5:**
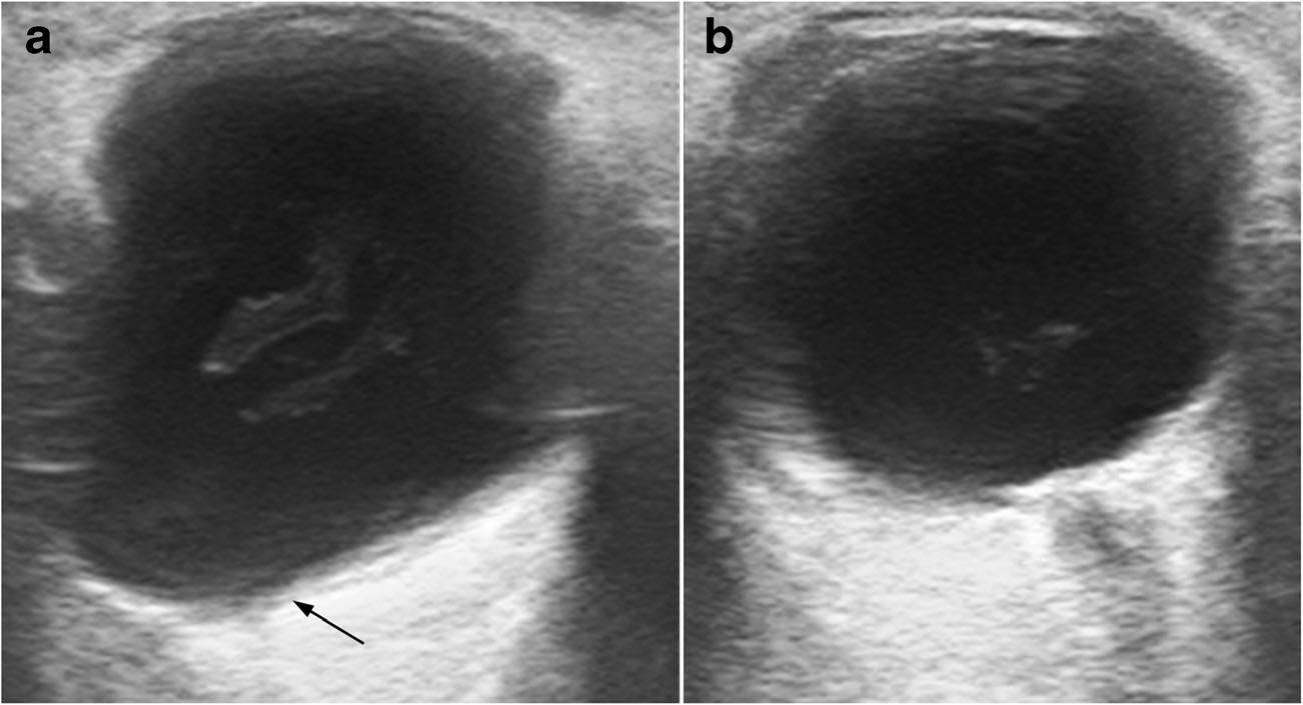
Comparative study in a woman with anisometropia in long-standing myopia in the right eye. **a**. Longitudinal US shows the lengthened anteroposterior axis of the right eye, with a pear-shaped posterior pole sacculation known as a staphyloma (*arrow*). **b** Compare with the normal left globe

In patients with long-term myopia, the anteroposterior axis of the globe is lengthened. The globe sometimes develops a thin wall, often manifesting as a pear-shaped sacculation of the posterior pole, also referred to as a posterior staphyloma, which can also occur secondary to glaucoma or trauma (Fig. 5) [7].

**Fig. 6:**
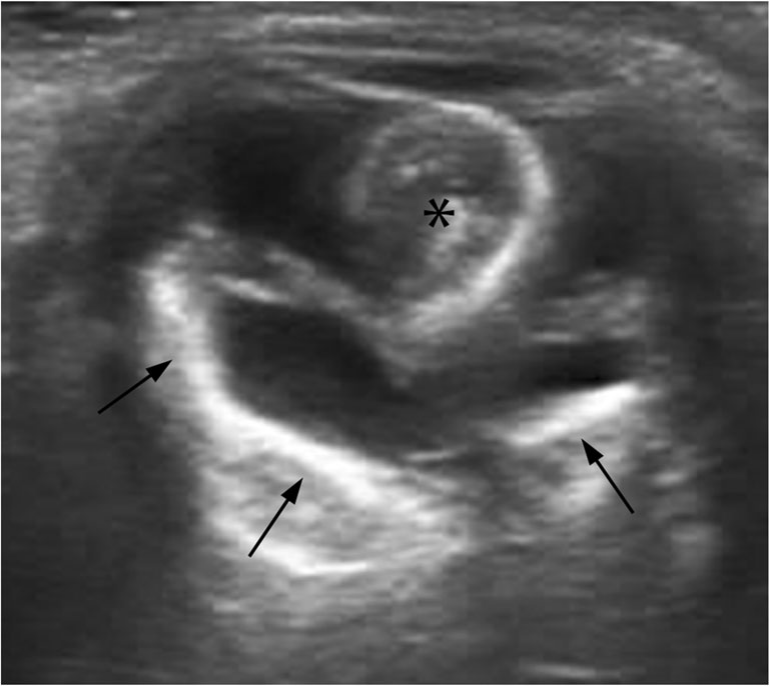
Phthisis bulbi in a 63-year-old man with a history of left ocular trauma during infancy. Axial US image shows a small, crenated, shrunken-looking ocular globe with calcified walls (*arrows*). Cataracts and partial lens dislocation (*asterisk*) are also seen

Phthisis bulbi refers to the end stage of many ocular disorders, commonly seen after trauma, failed surgery, and congenital ocular abnormalities. On US, the eye looks shrunken, usually with calcified walls and hyperechoic fibrous tracts from the retina to the posterior lens that can result in retinal detachment (Fig. 6) [7, 17].

### Lens pathology

**Fig. 7:**
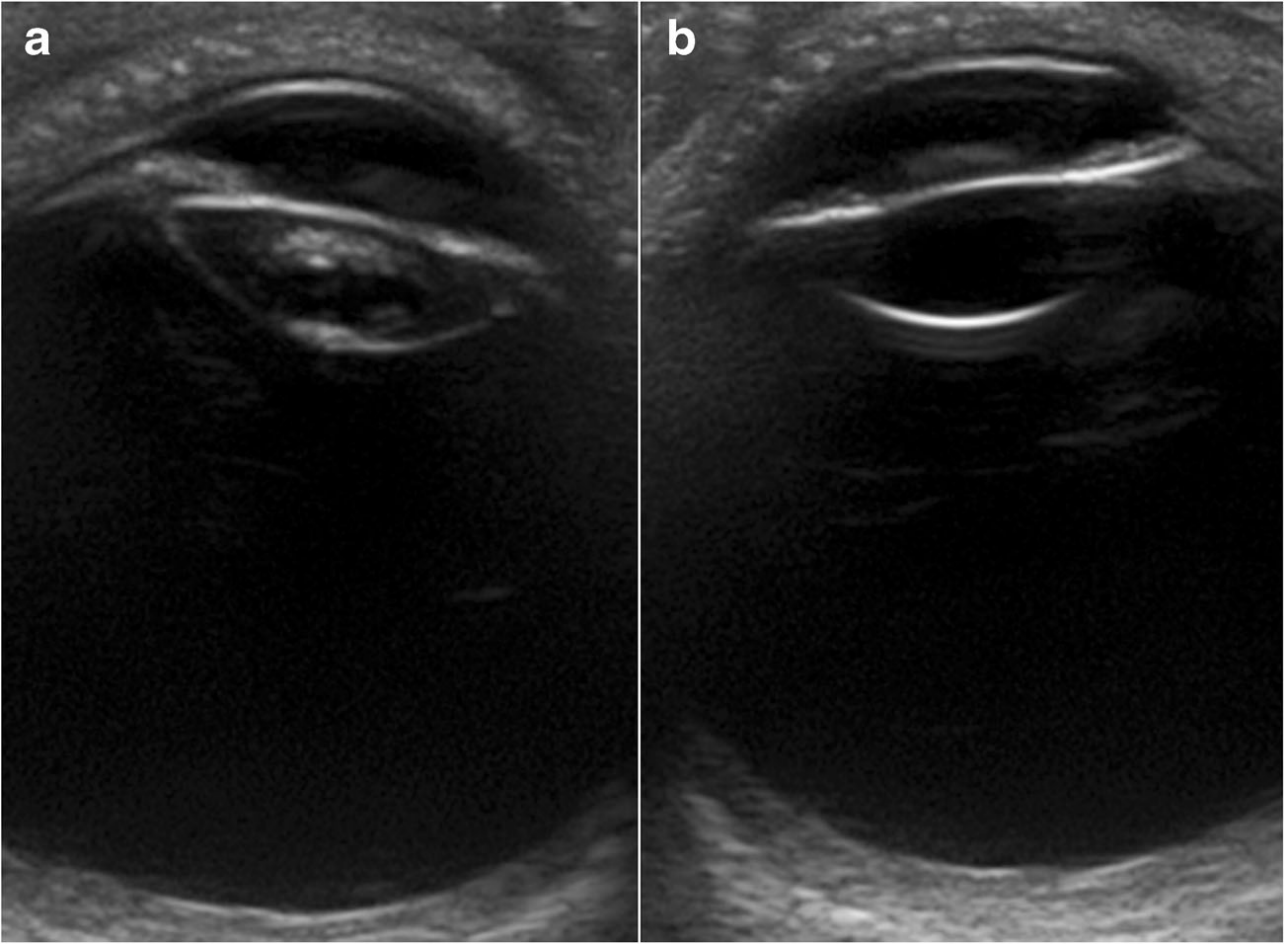
Unilateral cataracts in a 73-year-old man. Compare the intralenticular echoes in the right eye (**a**) with the normal appearance of the lens in the left eye (**b**)

**Fig. 8:**
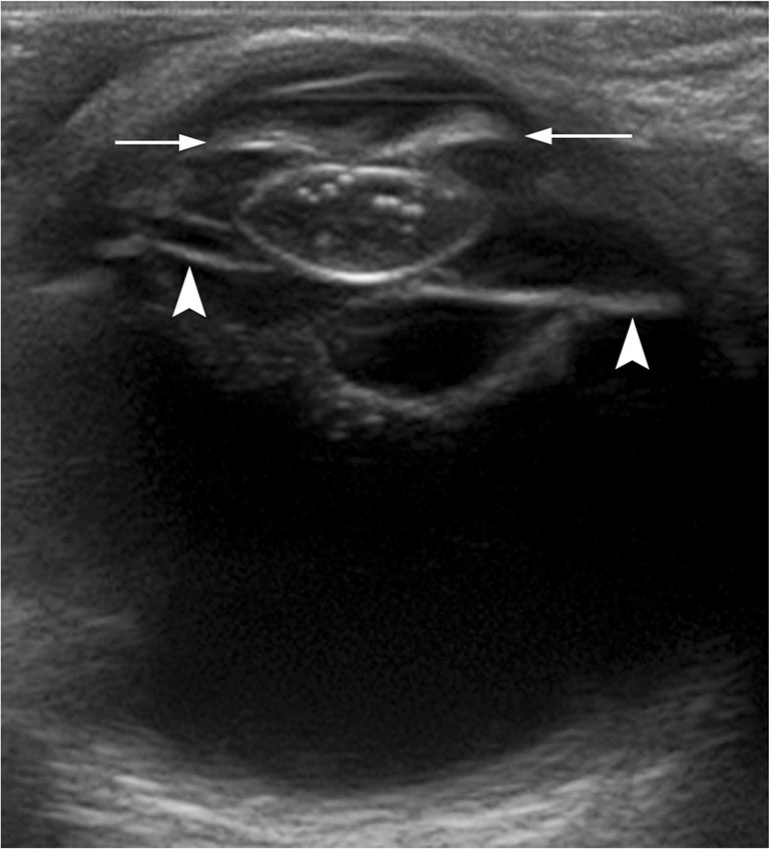
A 74-year-old man with a history of ocular trauma 20 years earlier. Axial US image of the right eye shows increased echogenicity of both walls of the lens, with intraocular echoes. There is also a synechia between the iris and the lens, causing the iris to curve forward (*arrows*)—the so-called iris bombe. This condition prevents the flow of aqueous humour from the posterior to the anterior chamber. Also note the thick posterior membranes between the lens and the vitreous (*arrowheads*)

Cataracts are a degenerative disease of the lens that is usually seen in older patients. They can also be congenital or occur secondary to trauma or infection. Ophthalmoscopy shows a white reflection with an opaque lens (leukocoria). In immature cataracts, scattered opacities are separated by clear zones (Fig. 7). In a complete cataract, the lens has a completely opaque cortex; on US it is seen as a hyperechoic structure (Fig. 8). Although cataract detection is not the primary aim of US, this technique is often routinely performed before mature cataract extraction in order to rule out possible contraindications to surgery, such as retinal detachment or tumours, that cannot be seen on the ophthalmoscopic examination because of the cataract and could influence the choice of treatment and prognosis [8, 12, 18].

**Fig. 9:**
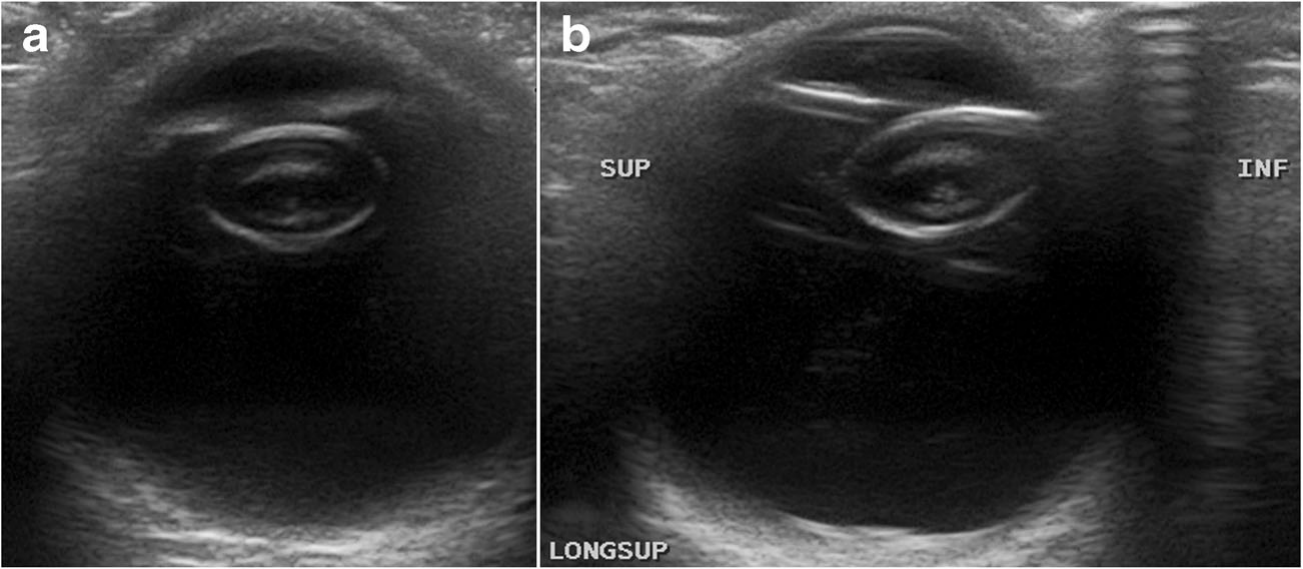
Partial lens dislocation in a 45-year-old man with a history of ocular trauma 2 years earlier. He developed a post-traumatic cataract, and physical examination detected an abnormal vibration or agitated motion of the iris during eye movements—the so-called iridodonesis (movie 1). **a** US examination with the patient in a supine position showed a globulous lens situated in a normal position, as well as cataracts. **b** US examination with the patient sitting upright revealed that the lens was detached at the 12 o’clock position. It is sometimes useful to perform US studies with the patient in different positions

**Fig. 10:**
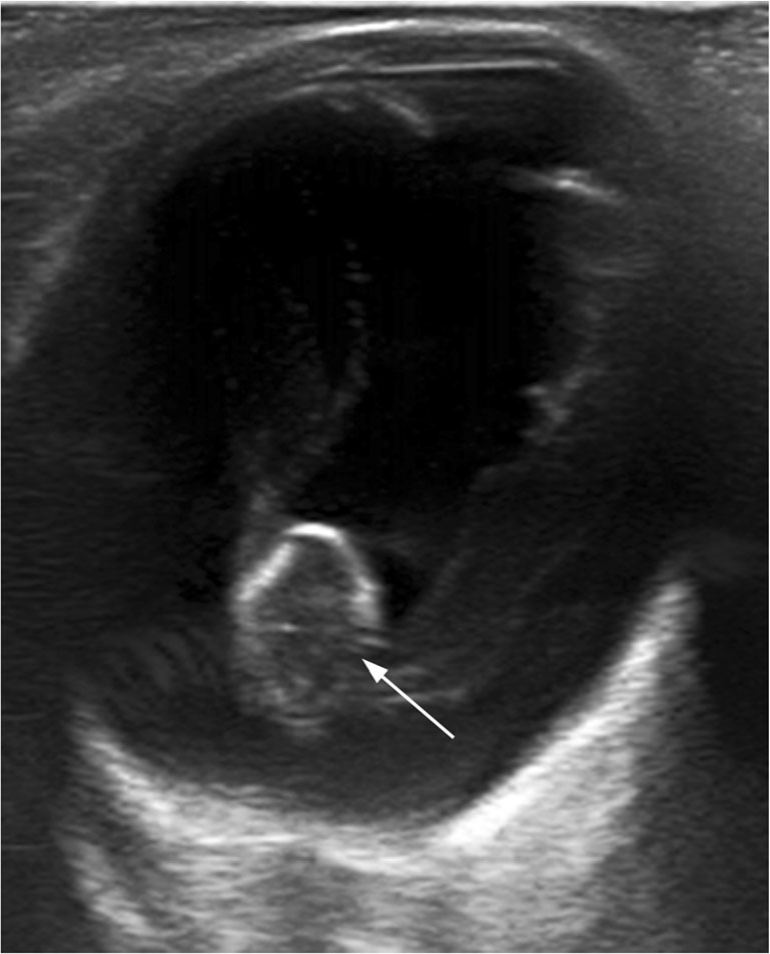
Complete lens dislocation in a man with a history of ocular trauma. Axial US image shows the dislocated lens (*arrow*), which lies entirely within the vitreous. Membranes from associated vitreous haemorrhage can also be seen

Ectopia lentis, a dislocation or malposition of the lens, is largely caused by ocular trauma, but it is also seen in collagen disorders such as Marfan and Ehlers-Danlos syndromes. There are two major types of dislocation: partial (subluxation) and complete. In partial dislocation, the lens remains partially attached to the ciliary body (Fig. 9). In a complete dislocation, the lens sinks in the vitreous body, lying over the retina, though it does move during dynamic examination (Fig. 10). A traumatic dislocation may be associated with a traumatic cataract and vitreous haemorrhage [5, 7].

Movie 1(MP4 2116 kb)

### Vitreous pathology

The vitreous is an acellular viscous fluid with 99 % water content and whose major molecular constituents are type 2 collagen fibrils and hyaluronic acid. Its low molecular and cellular content is essential for the maintenance of transparency. Its composition changes with age, so its appearance also changes [19]. In the US examination, increasing the gain shows a few low-amplitude punctate and linear mobile echoes floating within the vitreous chamber, often referred to as “floaters”. This finding is more evident in the dynamic study [5, 8].

**Fig. 11:**
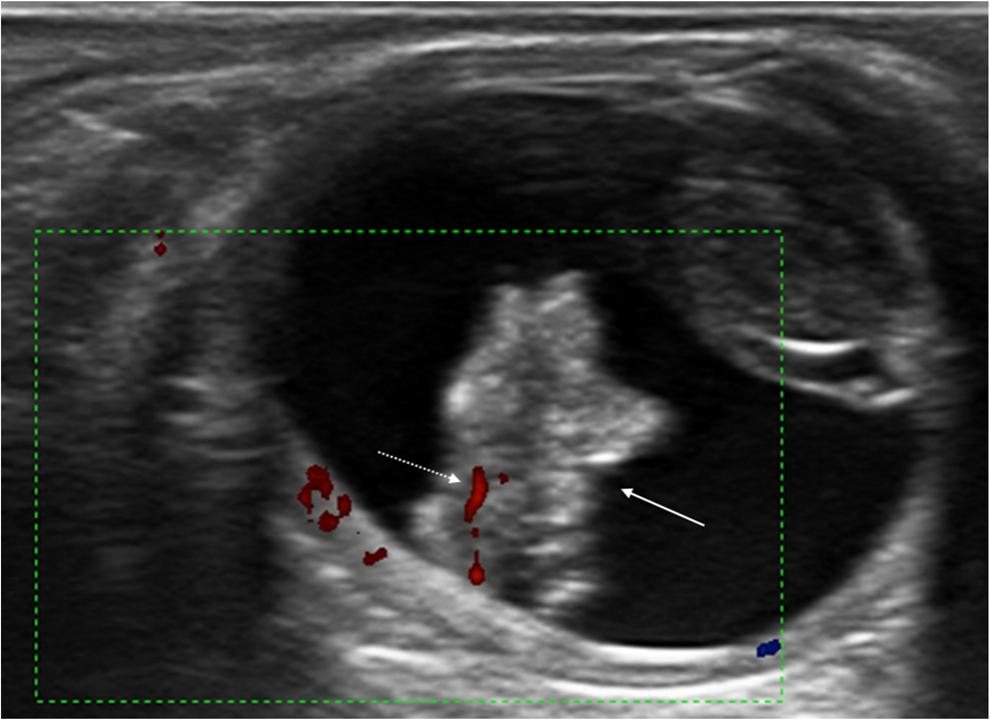
Ocular US in a 25-year-old woman with trisomy 21 and persistent hyperplastic primary vitreous. Axial US image shows a right eye that is smaller than normal, with a linear echogenic tract extending from the posterior surface of the lens to the posterior ocular wall representing the fibrovascular remnant (*arrow*). Colour Doppler shows arterial blood flow within the band that corresponds to persistence of the hyaloid artery (*dotted arrow*). Note also the cataract

Persistent hyperplastic primary vitreous is a congenital developmental anomaly resulting from the failure of the embryological primary vitreous and hyaloid vasculature to regress. It is typically a unilateral process without associated systemic findings, usually idiopathic, although it is sometimes found in rare systemic syndromes and genetic disorders. Less than 10 % of cases are bilateral. A persistent hyaloid artery may also be present. Persistent hyperplastic primary vitreous is often associated with microphthalmia and congenital cataracts. Retinal detachment owing to vitreoretinal traction is seen in 30 % of cases. US shows a hyperechoic band extending from the posterior surface of the lens to the posterior pole of the globe. This band represents a fibrous tract that corresponds to embryonic remnants of the primary vitreous, which may contain the hyaloid artery (Fig. 11) [16, 20].

**Fig. 12:**
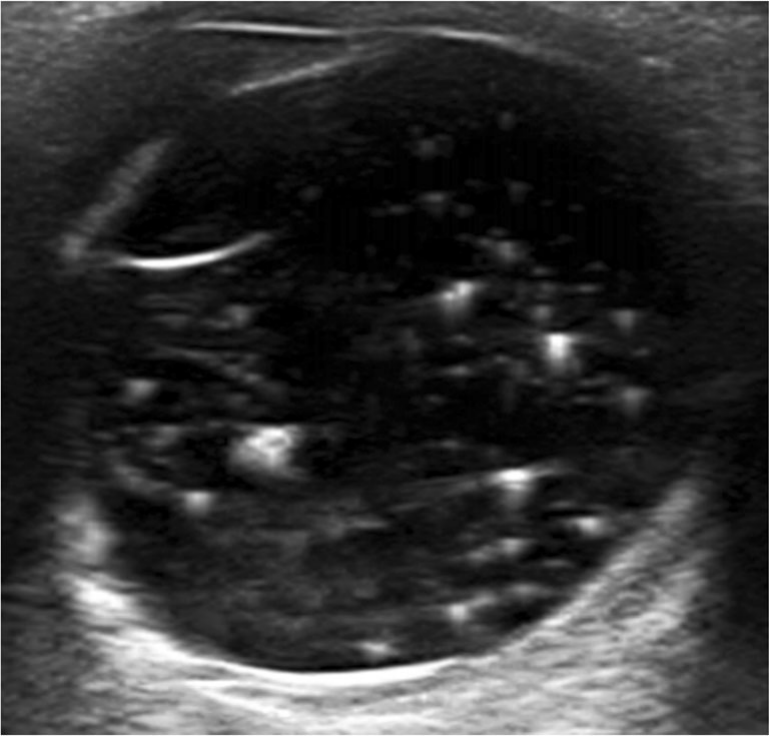
Asteroid hyalosis. Axial US in a 58 year–old man without visual disturbances. Note the incidental presence within the vitreous of numerous small hyperechoic echoes with comet-tail artefacts

Asteroid hyalosis is a degenerative condition of the eye of unknown origin; it is characterized by minute opacities due to fatty calcium soap deposits in the vitreous body. It is usually unilateral. Asteroid hyalosis rarely produces significant visual impairment, but it can obscure the examiner’s view of the fundus. At US, multiple small hyperechoic mobile echoes are seen in the vitreous. The deposits produce a sparkling appearance on real-time US reminiscent of the particles in a snow globe (Fig. 12; movie 2) [1, 3, 8].

Movie 2(MP4 4437 kb)

**Fig. 13:**
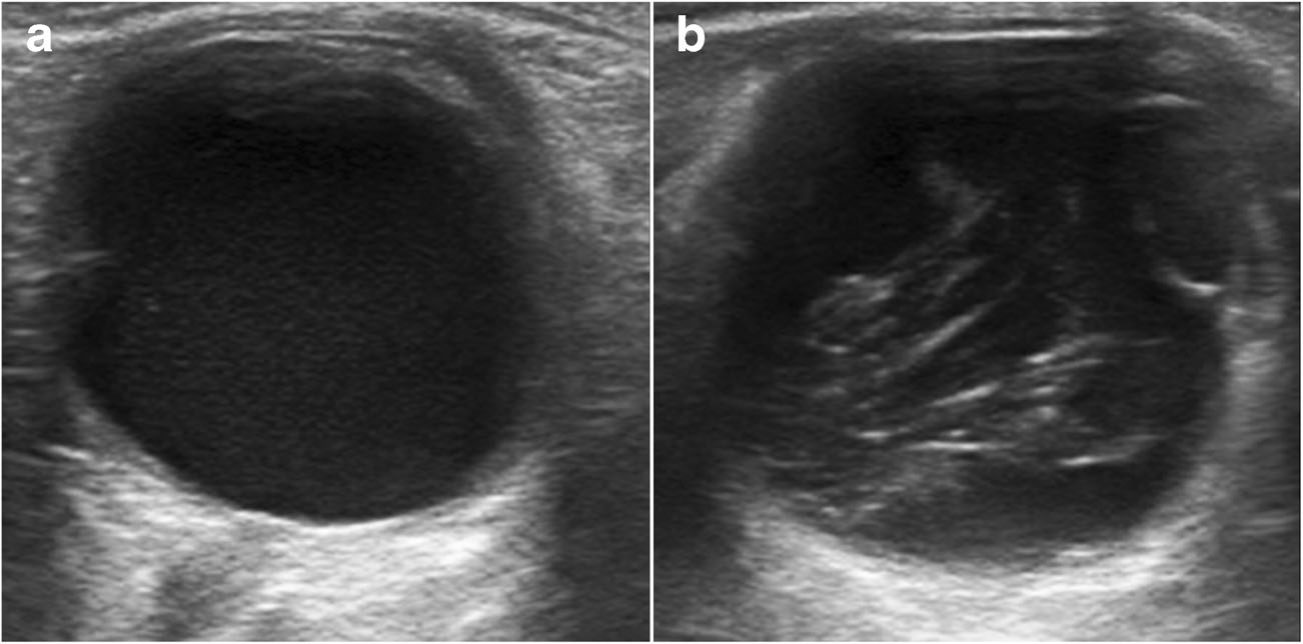
Acute and chronic vitreous haemorrhage in two different patients. **a** 72-year-old man with history of diabetes mellitus who presented with sudden blurred vision due to acute vitreous haemorrhage. At US, subtle echogenic material that obscured the ophthalmoscopic examination is seen floating within the vitreous. **b** Axial US image in a 58-year-old man with a history of vitreous haemorrhage 4 months earlier shows echogenic bands in the vitreous representing fibrous membranes, characteristic of chronic vitreous haemorrhage. The patient went on to undergo vitrectomy

Vitreous haemorrhage may occur in vasoproliferative diseases (diabetic retinopathy), retinal tears, posterior vitreous detachment, retinal macroaneurysms, age-related macular degeneration, and trauma, among others. The patient complains of “black rain” and of reduced visual acuity. Vitreous haemorrhage frequently obscures the retina from funduscopic visualization, and as such it is one of the most common indications for ocular US. Vitreous haemorrhages are followed up at 2–4-week intervals to check for clearing or for membrane formation. On US, the appearance of vitreous haemorrhage varies with the severity and the phase of the bleeding. In the early phase (up to a few days), US signs of haemorrhage may be very subtle, with only a few very low-amplitude echoes (Fig. 13). As the haemorrhage matures and organizes, fibrinous vitreous membranes may develop. These membranes are initially very mobile on dynamic US, but stiffen over time. Although they can mimic retinal detachments, fibrinous vitreous membranes are usually finer than the detached retina, move with the vitreous gel on dynamic scanning, and lack an anatomic attachment to the optic disc (Fig. 13). The membranes may retract, and if vitreoretinal proliferations are present, a tractional retinal detachment may occur. Vitreoretinal proliferations may require vitrectomy [3, 6, 8, 21].

**Fig. 14:**
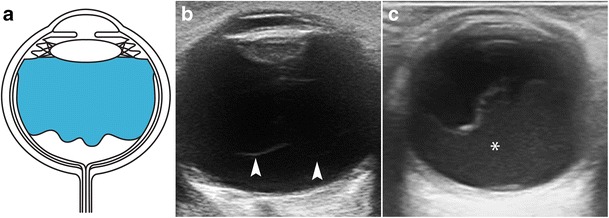
Posterior vitreous detachment (PVD) in two patients. **a**, **b** Illustration and US image correlation showing a PVD. Note the undulating appearance of the posterior vitreous that is better identified on dynamic scanning (*arrowheads*). **c** Axial US in a 67-year-old man with blurred vision. Note the fine echoes in the retrohyaloid space (*asterisk*), consistent with retrohyaloid haemorrhage

Posterior vitreous detachment is an-age related phenomenon in which the posterior vitreous capsule or hyaloid detaches from the underlying retina. On dynamic US examination, the posterior detached vitreous is seen as an undulating membrane that moves freely and should swirl away from the region of the optic disc in cases of complete posterior vitreous detachment (Fig. 14; movie 3). These findings are highly characteristic. Vitreoretinal adhesions can cause retinal tears or avulsion of a peripheral blood vessel, resulting in vitreous and retrohyaloid haemorrhage (Fig. 14; movie 4). Posterior vitreous detachment is not always associated with symptoms, and is sometimes encountered as an incidental finding [1, 6, 8].

Movie 3(MP4 2476 kb)Movie 4(MP4 2612 kb)

### Retinal pathology

**Fig. 15:**
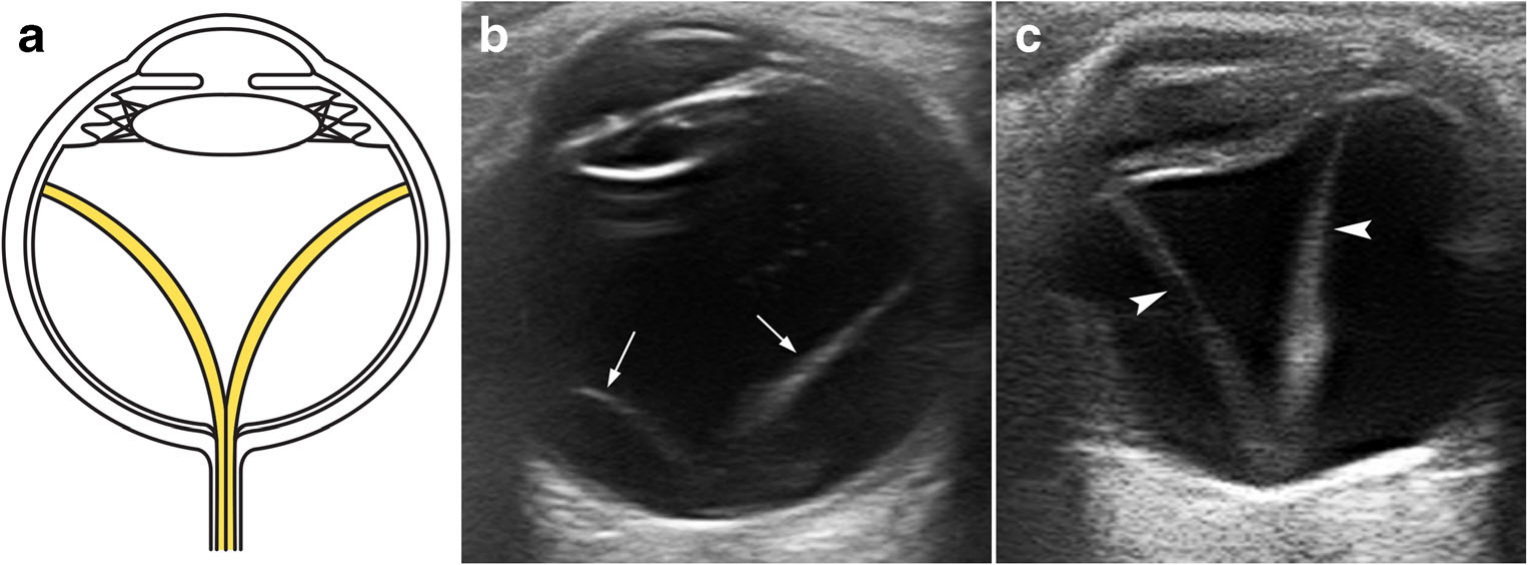
Retinal detachment. **a** Diagram illustrating total retinal detachment. Note the anatomic retinal attachment points. **b** Axial US image in a 45-year-old man with Marfan syndrome: two echogenic lines form an acute angle, with the “V” shape characteristic of an acute retinal detachment. At this stage, the retinal leaves are thin and mobile (*arrows*). This condition requires surgical treatment. **c** 75-year-old woman with chronic retinal detachment. At this stage, the retinal leaves look thick and rigid, adopting a funnel-shaped configuration (*arrowheads*); this condition is not amenable to surgical treatment

**Fig. 16:**
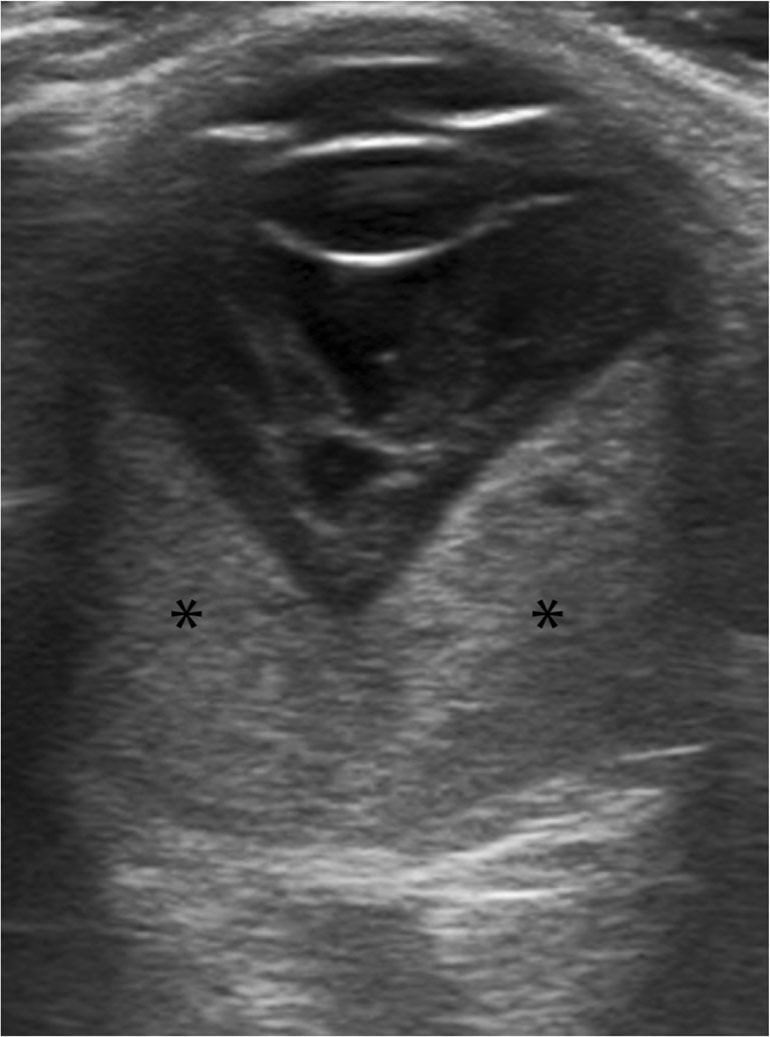
Subretinal haemorrhage. Axial US shows the subretinal space (*asterisks*) filled with an echogenic material consistent with blood

**Fig. 17:**
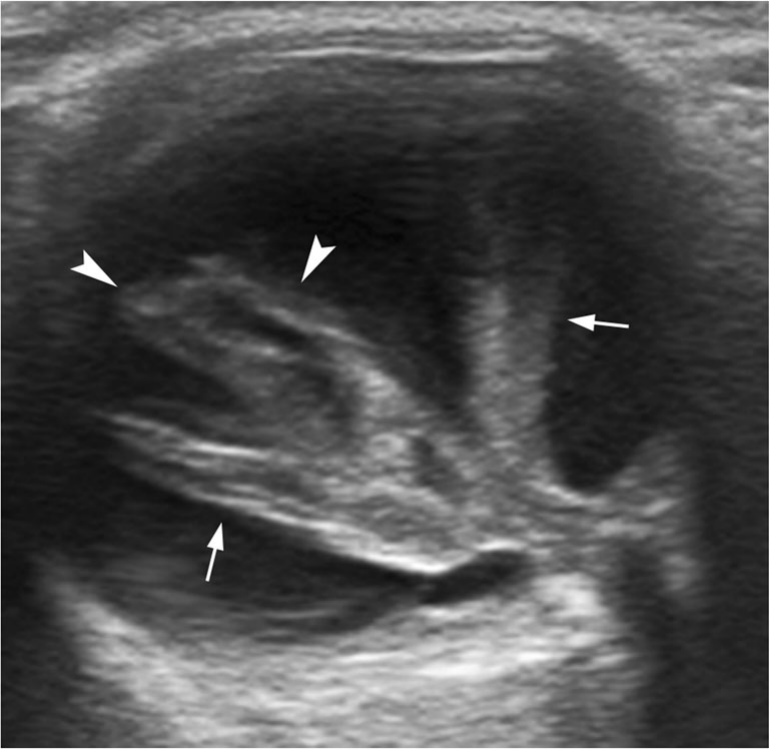
An 83-year-old woman with uncontrolled diabetes and a history of proliferative diabetic retinopathy and vision loss in the left eye. Axial US image shows thick membranes (*arrowheads*) in the vitreous and a tractional total chronic retinal detachment (*arrows*)

**Fig. 18:**
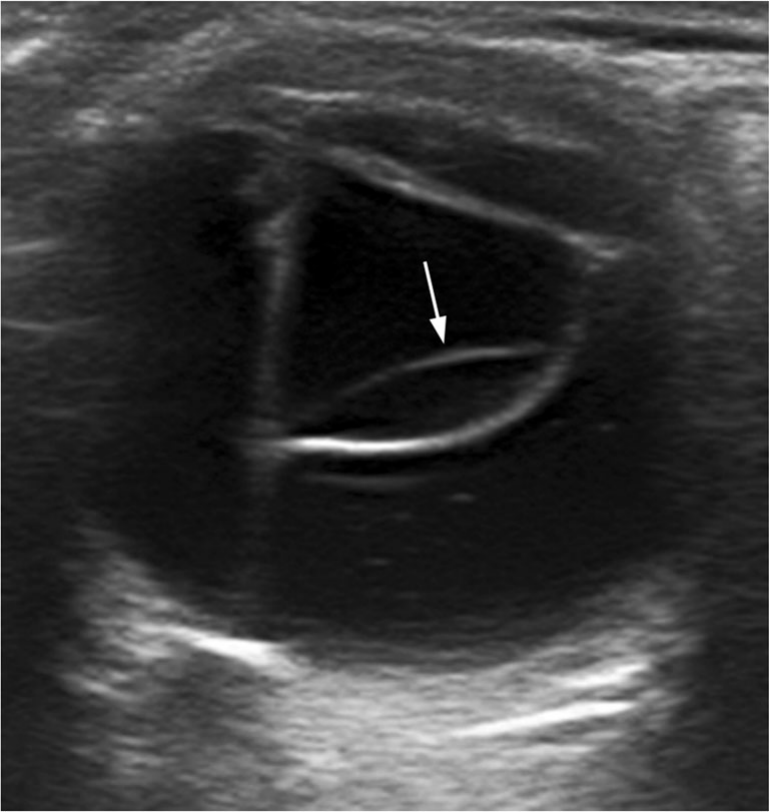
Woman with chronic right retinal detachment who developed a retinal cyst (*arrow*) on the nasal side

Retinal detachment refers to the separation of the inner sensory layer of the retina from the outer pigmented layer [18]. Retinal detachments are classified into three types, depending on the underlying mechanism: rhegmatogenous retinal detachment results from a retinal tear; tractional retinal detachment results from vitreoretinal traction due to contracting membranes; and exudative retinal detachment results from blood, exudative fluid, or a lesion in the subretinal space [7, 12]. Detachment is classified as total, partial, or focal, depending on its extension. On US, a total retinal detachment appears as a “V” shape in the vitreous cavity, because the retina remains firmly attached to the ora serrata anteriorly and to the optic nerve head posteriorly (Fig. 15). In partial detachment, a linear echogenic membrane can be seen, usually extending to the optic nerve head, but not across it. The point of fixation at the optic nerve head is a useful feature for differentiating between retinal detachment and vitreous membrane [18]. Retinal detachment is sometimes associated with subretinal haemorrhage (Fig. 16). In the acute setting, the retinal leaves look thin and mobile on dynamic scanning, whereas chronic detachments display thicker, echogenic leaves that are not mobile on dynamic examination. Chronic detachment is often seen as a rigid “triangle sign” (Fig. 17) [5, 12, 21]. Eventually, cysts may form within the retinal leaves in long-standing RD (Fig. 18) [8].

**Fig. 19:**
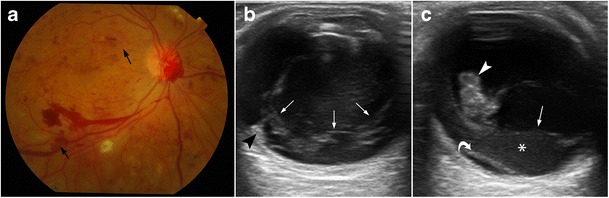
Proliferative diabetic retinopathy (PDR). **a** Fundoscopy image shows extensive neovascularization (*arrows*), the hallmark of PDR. **b** US images in a 72-year-old diabetic man with PDR and a history of recurrent vitreous haemorrhage. Note the vitreous bands due to chronic vitreous haemorrhage (*arrows*) and preretinal fibrovascular membranes (*arrowhead*). **c** At the 3-month follow-up, the fibrovascular tissue has grown into the vitreous cavity (*arrowhead*), and the patient has developed posterior vitreous detachment (*arrow*), retrohyaloid haemorrhage (*asterisk*), and focal retinal detachment (*curved arrow*). He subsequently underwent vitrectomy and panretinal laser photocoagulation

Proliferative diabetic retinopathy (PDR), which occurs in diabetic patients with poor glucose control, is one of the most common causes of vitreous haemorrhage. It manifests as progressive changes in the eye microvasculature. PDR-related complications are better detected by fundoscopy and fluorescein angiogram, but some can be identified using US when opacification of the transparent media precludes visualization by fundoscopy. US can show vitreous haemorrhage, retrohyaloid haemorrhage, and occasionally focal thickening in the macular area that could occur secondary to subretinal haemorrhage or diabetic macular oedema, mimicking a tumour [1]. In advanced stages of the disease, preretinal membranes can form, which may contract and cause retinal detachment (Fig. 19) [1, 22].

### Choroidal pathology

**Fig. 20:**
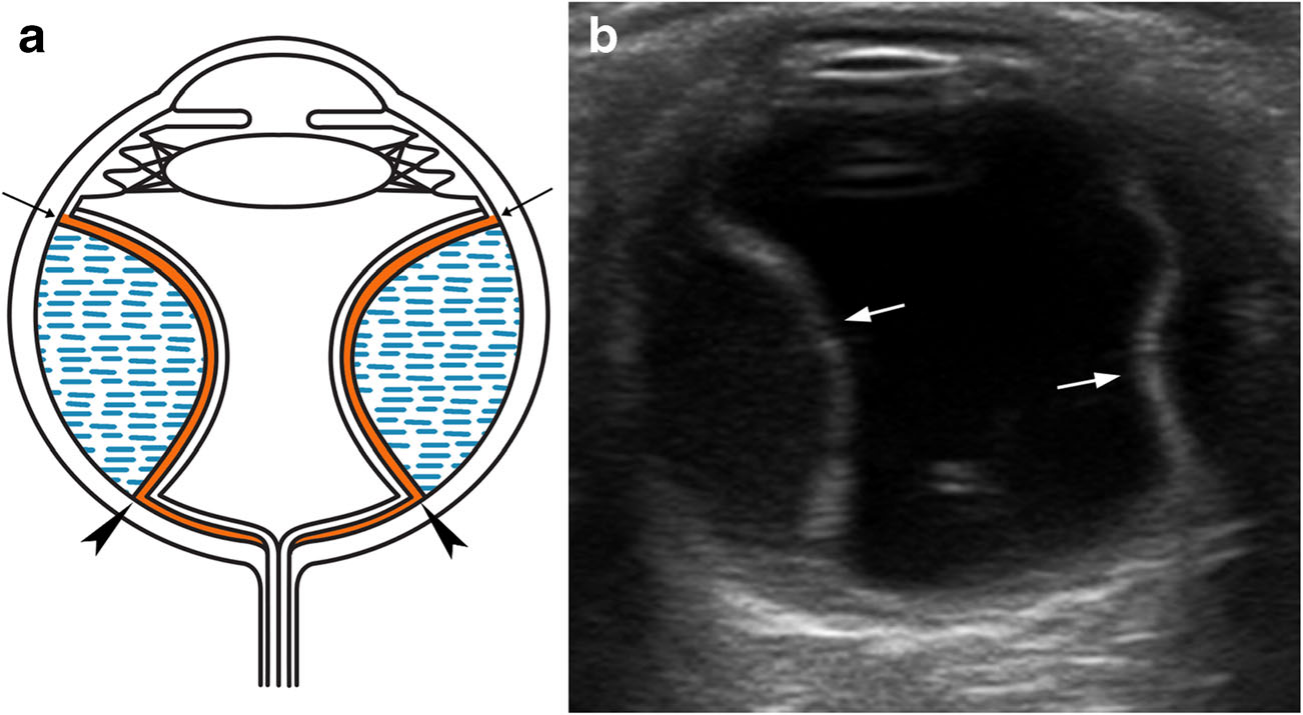
Choroidal detachment. **a** Diagram showing typical bilateral exudative choroidal detachment, with two convex rigid lines protruding into the vitreous, extending from the scleral spur near the ciliary body (*arrows*) to the level of the exit foramina of the vortex veins (*arrowheads*), at some distance from the papilla. **b** US image in a 69-year-old woman with glaucoma in the right eye 1 day after cataract surgery shows two lines ballooning into the vitreous, demonstrating an exudative choroidal detachment (*arrows*)

Choroidal detachment, also known as uveal effusion, is less common than retinal detachment, and is caused by the accumulation of fluid in the potential space located between the choroid and the sclera [12]. It may result from trauma, surgery for glaucoma, lens extraction for cataracts, or hypotony of any cause [23]. At US, the choroid balloons into the eye and protrudes convexly into the vitreous. The bands visible in the choroidal detachment are typically thick and rigid; they end at the level of the exit foramina of the vortex veins and do not extend to the optic disc (Fig. 20). Arterial flow can be seen in these thick membranes. Subchoroidal haemorrhage may be associated. In these cases, low- or medium-level echoes can be seen between the choroid and the sclera [1, 8, 12, 24].

Choroidal tumours include benign and malignant conditions. Most ophthalmic malignancies fall into one of these three groups: uveal melanoma (70.4 %), retinoblastoma (9.8 %), and metastases (9.2 %) [25]. Both primary and metastatic tumours typically present with visual disturbances or marked vision loss. They may also be discovered incidentally on imaging for monocular disease. Ophthalmologic examination may demonstrate a mass lesion, with or without retinal detachment or vitreous haemorrhage.

**Fig. 21:**
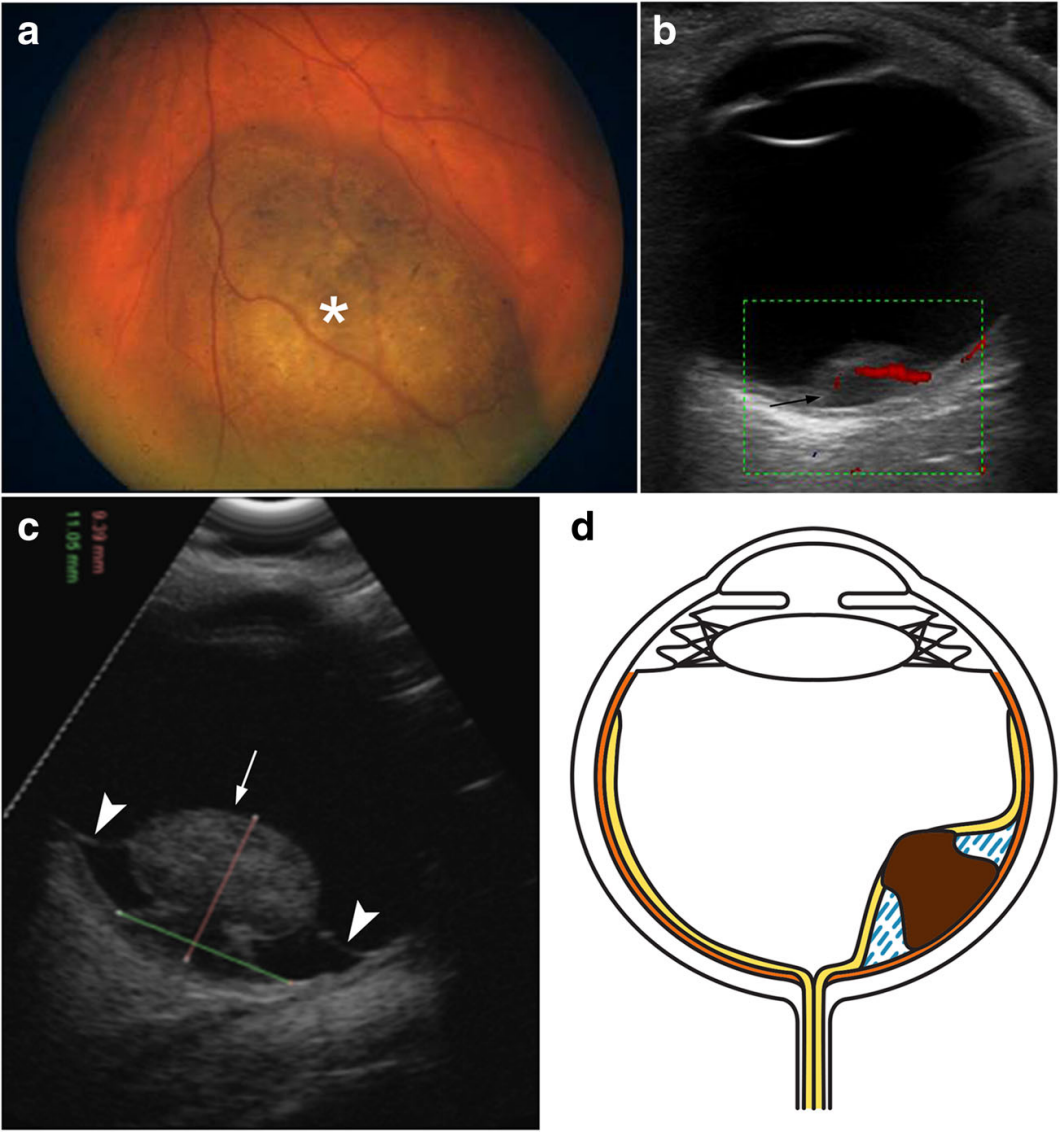
Choroidal melanoma in two patients. **a** 62-year-old man with a 6-month history of progressive vision loss in the right eye. Fundoscopy image shows a pigmented mass in the inferior nasal arcade of the right eye (*asterisk*). **b** Colour Doppler US image shows a dome-shaped hypoechoic posterior mass with a smooth surface and choroidal excavation underneath (*arrow*), as well as internal blood flow. **c**, **d** US obtained in a 54-year-old man shows a mushroom-shaped mass related to a rupture of Bruch’s membrane. Note the peritumoral serous retinal detachment (*arrowheads*) induced by the growth of the tumour, as illustrated in the diagram

Ocular melanoma arises from the melanocytes of the outer layers of the choroid. Small melanomas are dome-shaped. In some cases, as the tumour grows, it breaks through Bruch’s membrane into the subretinal space, forming a neck or stalk and acquiring a mushroom shape, which is a pathognomonic feature of ocular melanoma. The tumour can grow, causing the retina to detach at the edges while remaining attached at the summit of the growth. Melanoma may grow progressively within the globe or it may extend outwards to the orbital tissues [25]. The cardinal US features of ocular melanoma include solid consistency, regular internal structure, dome shape (in most cases) or mushroom shape (in few cases), low to medium echogenicity, internal blood flow at the base, and choroidal excavation under the mass (Fig. 21) [1, 26–28].

Ocular metastases usually come from breast, lung, or kidney tumours [25]. At US, metastases appear on the posterior wall as a flat mass with an irregular surface, and they usually appear hyperechoic compared with melanoma. Occasionally, multiple and bilateral metastases are present [3, 7, 29].

Choroidal nevi are the most common benign intraocular tumours, affecting 4 to 8 % of the population [30]. However, they sometimes cause vision loss or visual field defects, and can (rarely) transform into malignant melanoma. At US, they are seen as flat echogenic lesions in the posterior wall; they typically have a regular internal structure with medium to high echogenicity. Their thinness makes it difficult to differentiate them from small choroidal melanomas.

**Fig. 22:**
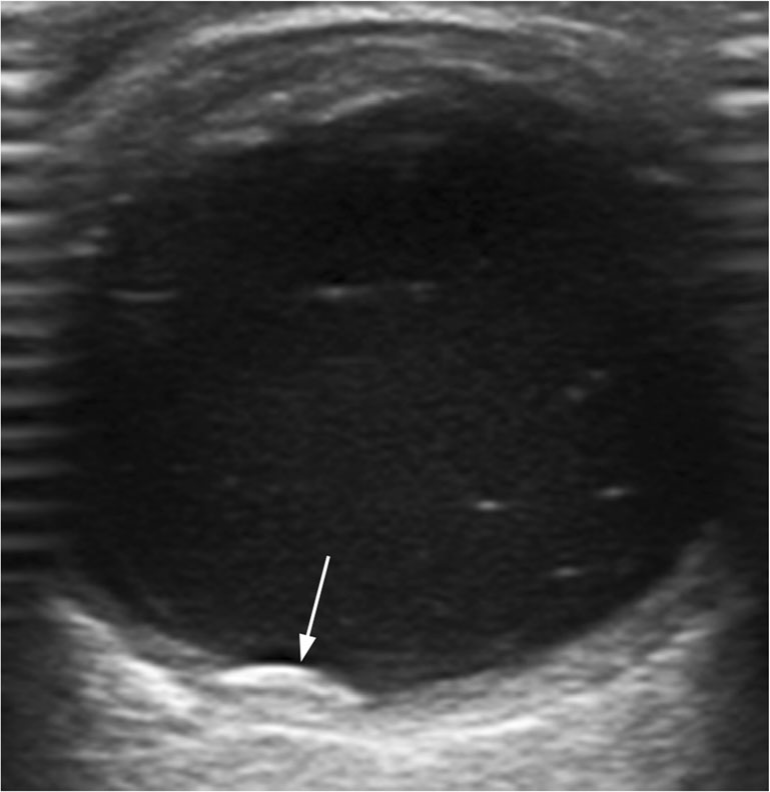
Choroidal hemangioma in a 45-year-old woman with choroidal hemangioma suspected at fundoscopy. Axial US shows a small homogeneous hyperechoic biconvex lesion (*arrow*) on the temporal side of the globe near the papilla

Choroidal hemangiomas are vascular malformations that usually present as solitary lesions, although they can present as large diffuse areas in the context of Sturge-Weber syndrome. Choroidal hemangiomas are frequently located in the macular region of the posterior pole. US shows a homogeneous biconvex echogenic mass with a highly echogenic regular internal structure, without calcifications (Fig. 22). Choroidal hemangiomas have low blood flow, so flow may not be evident on Doppler US [7, 16].

### Tumour mimics

Entities that can mimic tumours on US include age-related macular degeneration (AMD), subretinal haemorrhages, and macular oedema resulting from diabetes, vascular occlusion, and inflammatory conditions.

**Fig. 23:**
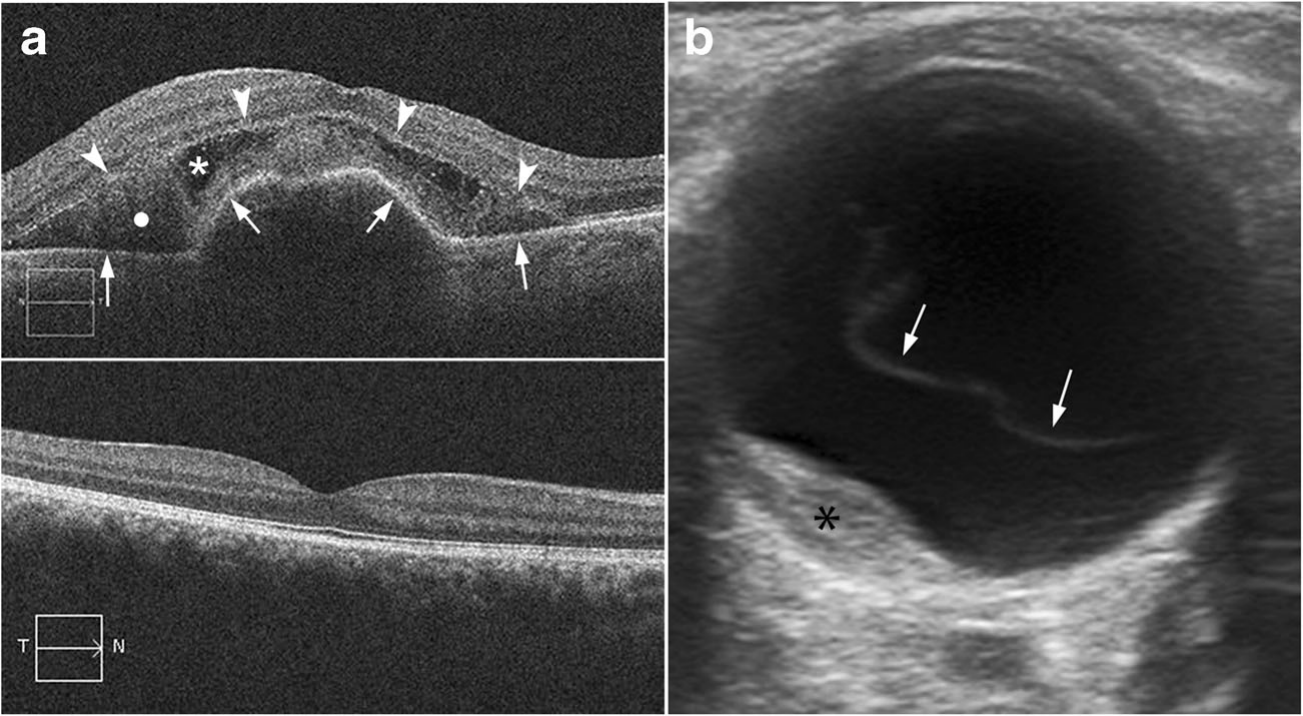
Neovascular age-related macular degeneration. **a** Top image: optical coherence tomography (OCT) image in a patient with active neovascular age-related macular degeneration (AMD) shows serous detachment of retinal pigment epithelium (*arrows*) and neurosensory retinal detachment (*arrowheads*). Note also the subretinal haemorrhage (*white circle*) and subretinal fluid (*white asterisk*). Bottom image: non-pathologic OCT in another patient. **b** 71-year-old man with progressive vision loss in the right eye. US shows a mass on the temporal side of the posterior wall next to the papilla (*asterisk*); note also the posterior vitreous detachment (*arrows*). Final diagnosis was subretinal haemorrhage due to choroidal neovessels in the context of neovascular AMD

AMD is a chronic disease that causes vision loss in the centre of the field of vision. There are two major types of AMD: the atrophic or nonexudative type and the neovascular or exudative type. In neovascular AMD, the neovessels may bleed or leak, producing intra- and subretinal exudate and subsequently healing by fibrosis [1, 31]. The diagnosis is reached clinically with the aid of ophthalmologic tools such as fluorescein angiography and optical coherence tomography (Fig. 23). US may demonstrate an elevated mass on the posterior wall at the macular area that could be mistaken for a tumour (Fig. 23). These lesions are caused by subretinal exudates or haemorrhages in the region of the macula [1].

### Optic disc pathology

**Fig. 24:**
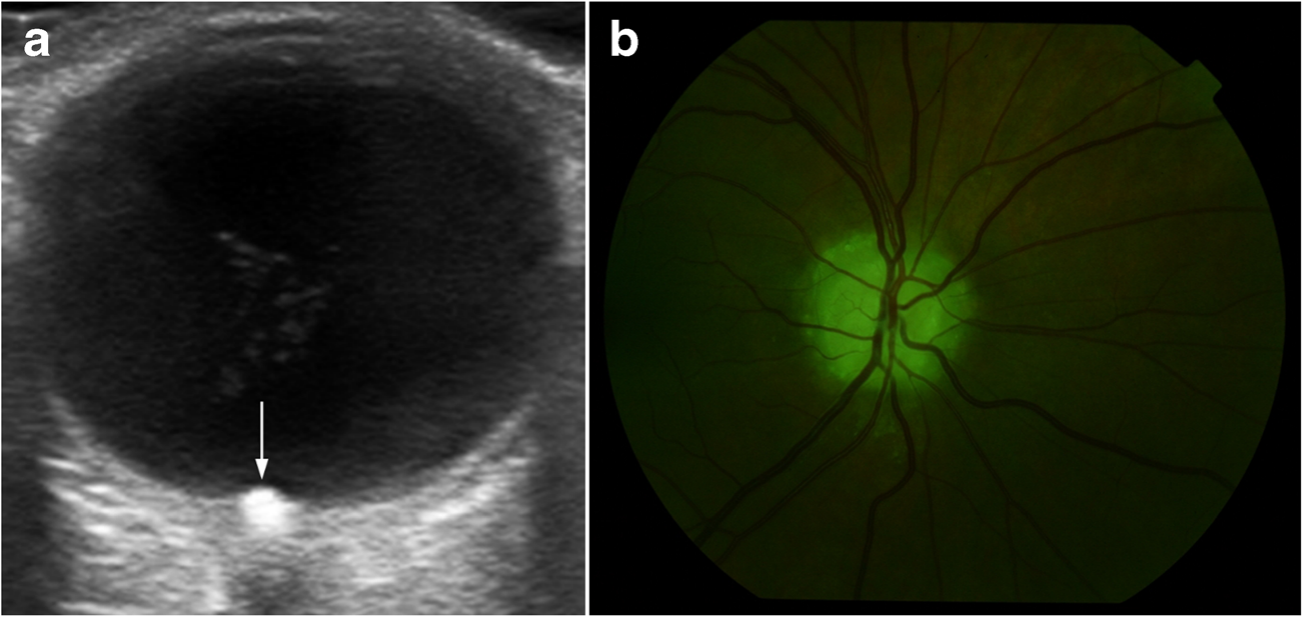
Optic disc drusen. **a** Axial US shows a typical calcified plaque at the optic disc (*arrow*). **b** Drusen typically exhibit autofluorescence

Optic disc drusen are calcified hyaline-like deposits in the optic nerve head. They occur in 0.4 to 20.4 % of the population and may be bilateral (67–91 %) [32]. They are usually asymptomatic, but may cause blurring or loss of vision. Optic disc drusen are easily diagnosed with fundoscopy if the classic finding of low-white, glistening hyaline deposits can be identified. Another distinguishing feature is their autofluorescence. Nevertheless, when they lie deep within the tissue of the optic nerve, they may mimic papilledema; US is useful in these cases, demonstrating an echogenic focus of variable size at the optic disc. If strongly calcified, they can have posterior shadowing (Fig. 24) [24].

### Post-surgical conditions

**Fig. 25:**
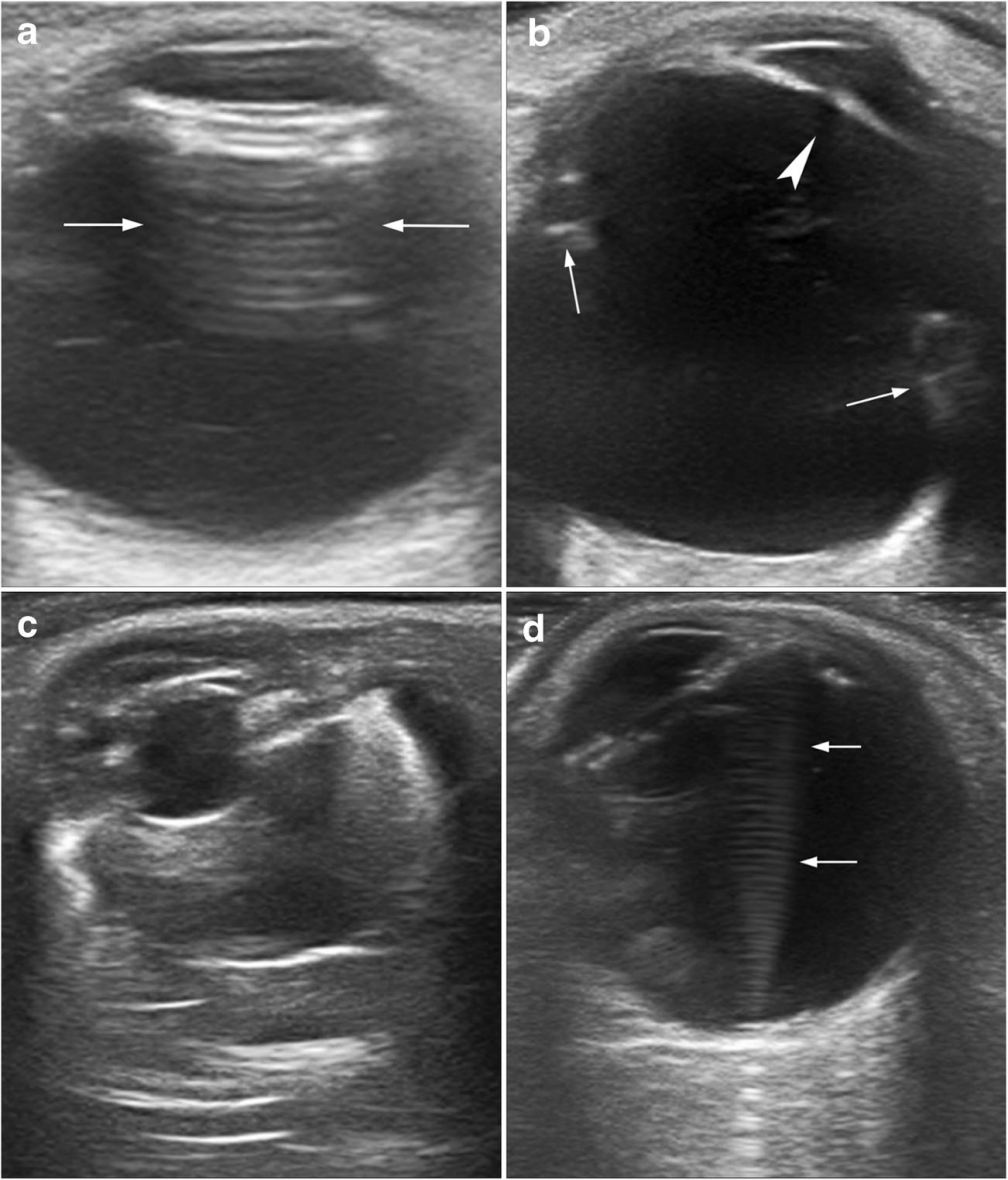
Post-surgical conditions. **a** Patient with pseudophakia with an intraocular lens. Note the reverberation artefact produced by the intraocular lens (*arrows*). **b** US appearance of scleral buckling (*arrows*) used to treat retinal detachment (RD). Note the absence of the lens in this aphakic patient (*arrowhead*). **c** US showing silicone oil injection and scleral buckling, with artefacts produced by the silicone. **d** Reverberation artefact caused by retention of a small volume of perfluorocarbon liquid in the vitreous cavity (*arrows*) in a man treated for RD

Pseudophakia refers to a condition in which an intraocular lens has been implanted after cataract extraction. US shows a highly echogenic flat or concave structure in the location of the lens, with a reverberation artefact behind the iris plane (Fig. 25) [7, 8].

Scleral buckling is a procedure to repair a retinal detachment by placing a band of material, usually Silastic, around the globe. The band causes some deformity of the globe, resulting in characteristic imaging features (Fig. 25) [8].

Silicone oil is sometimes instilled into the vitreous chamber to reduce retinal detachment. On US, it produces severe artefacts that prevent adequate evaluation of the posterior ocular segment (Fig. 25) [8].

Perfluorocarbon liquids (PFCL) are used in vitreoretinal surgery for various purposes, including the repositioning and fixing of a detached retina. These chemical compounds should be completely removed at the end of the surgery, as they can lead to complications. At US, PFCL retention is seen as echogenic images with marked reverberation artefacts (Fig. 25) [33].

Intraocular air and gas may be seen in the vitreous chamber immediately after surgery. It is highly echogenic, making it difficult to visualize the posterior segment. Turning the patient’s head to one side can displace the bubbles [8, 21].

## Conclusion

The eye is an organ ideally suited to US imaging. Knowledge about the anatomy, pathology, and US signs, together with a systematic approach, can provide useful diagnostic information. US has the advantage of being widely available, noninvasive, quick, and cost-effective. Modern multipurpose US scanners with high-frequency small parts probes are useful for ocular US. Dynamic and colour Doppler studies also provide valuable information. A multidisciplinary approach, especially in collaboration with ophthalmologists, is vital. Greater knowledge of ocular US will allow radiologists to improve the usefulness of this technique in the clinical setting.

## Electronic supplementary material

Below is the link to the electronic supplementary material.
